# Genetic diversity of *Ehrlichia ruminantium* field strains from selected farms in South Africa

**DOI:** 10.4102/ojvr.v87i1.1741

**Published:** 2020-06-25

**Authors:** Helena C. Steyn, Alri Pretorius

**Affiliations:** 1 Vaccine Development and Diagnostics, Onderstepoort Veterinary Research, Pretoria, South Africa

**Keywords:** *Ehrlichia ruminantium*, molecular epidemiology, phylogenetic analyses, qpCS20 real-time PCR, subtractive hybridisation polymorphic ORFs

## Abstract

Heartwater is a tick-borne disease caused by the intracellular rickettsial parasite *Ehrlichia ruminantium* and transmitted by *Amblyomma hebraeum* ticks. Heartwater is problematic in endemic areas because it causes high mortality in ruminants and leads to economic losses that threaten productivity and food security. This may indicate that there is augmented genetic diversity in the field, which may result in isolates that are more virulent than the Ball3 and Welgevonden isolates. The genetic diversity of *E. ruminantium* was investigated in this study, focussing on the pCS20 gene region and four polymorphic open reading frames (ORFs) identified by subtractive hybridisation. The 16S ribosomal ribonucleic acid gene confirmed *E. ruminantium* in brain, blood and tick genomic deoxyribonucleic acid samples (*n* = 3792) collected from 122 farms that were randomly selected from seven provinces of South Africa where heartwater is endemic. The conserved *E. ruminantium* pCS20 quantitative polymerase chain reaction (qPCR) assay was used to scan all collected field samples. A total of 433 samples tested positive with the qPCR using the pCS20 gene region, of which 167 were sequenced. The known stocks and field samples were analysed, and phylogenetic trees were generated from consensus sequences. A total of 25 new clades were identified; of these, nine isolates from infected blood could be propagated in cell cultures. These clades were not geographically confined to a certain area but were distributed amongst heartwater-endemic areas in South Africa. Thus, the knowledge of strain diversity of *E. ruminantium* is essential for control of heartwater and provides a basis for further vaccine development.

## Introduction

Heartwater is a tick-borne disease caused by the obligate intracellular rickettsial parasite, *Ehrlichia ruminantium. Amblyomma hebraeum* tick species transmit the *E. ruminantium* pathogens to livestock and wild ruminants. Heartwater is a major problem in the endemic areas of South Africa (SA) because it causes mortality in cattle, sheep and goats, especially the Angora goats that supply the mohair industry, and is responsible for high economic losses in both communal farming and the commercial sectors (Allsopp 2010). Molecular detection of *E. ruminantium* in animals and ticks focussed on the following genes or regions: the pCS20 region (Steyn et al. 2008; Van Heerden et al. 2004), the 16S ribonucleic acid (RNA) gene sequence (Allsopp et al. 1997) and the *map*1 gene family (Allsopp et al. 2001; Barbet, Byrom & Mahan 2009; Faburay et al. 2008). Other methods of detection include loop-mediated isothermal amplification (LAMP) (Nakao et al. 2010), multiple-locus variable tandem repeats (MLVA) (Pilet et al. 2012) and multi-locus sequence typing (MLST) (Adakal et al. 2009; Nakao et al. 2011). However, there is not a single method/gene that provides the full picture of genetic diversity of *E. ruminantium*.

Genetic diversity amongst *E. ruminantium* isolates has been confirmed in previous epidemiological studies using the *map*1 gene family with known strains and field isolates in infected sheep and cattle from different ecological heartwater-endemic origins in Africa. Some of these isolates were from Zimbabwe (Kwekwe, Crystal Springs, Plumtree and Palm River), Botswana (Sunnyside), SA (Welgevonden), Nigeria (Nigeria D225), Senegal (Senegal), Sudan (Um-Banein) and Caribbean (Antiqua, Gardel) (Allsopp et al. 2001; Faburay et al. 2008; Reddy et al. 1996). The *map*1-2 paralog showed the greatest diversity (79%) in the *map*1 gene family while the rest were more conserved (98% – 99%) (Barbet et al. 2009). The Zimbabwe stains, Highway, Crystal Springs and Nyatsanga, were closely related while the Senegal and Antiqua strains were identical to each other.

Currently, we have 17 *E. ruminantium* reference strains from different geographical origins in SA, sub-Saharan Africa and the Caribbean islands propagated in cell culture (Allsopp, Bezuidenhout & Prozesky 2005). In spite of identifying additional isolates, including the Crystal Springs stock (Zimbabwe), isolated from a dog (Allsopp & Allsopp 2001), the Breed strain isolated from an Angora goat (Du Plessis et al. 1983), Rietgat (Mahan et al. 1998), Skukuza (Peter et al. 1999) and Warmbath isolates (Mahan et al. 1998), there is little knowledge of the current diversity within *E. ruminantium* in SA. This highlights the need for an epidemiological survey in SA to determine the genetic diversity and occurrence of the pathogens in the field.

Genomic deoxyribonucleic acid (DNA) from these 17 reference strains were used for sequence analysis (pCS20 region, 16S ribosomal RNA [16S rRNA], *glt*A, *gro*EL, *fts*Z, *sod*B, *nuo*B and *map*1) to demonstrate the extensive recombination that occurs between strains and to distinguish one strain from another by phylogenetic analysis (Allsopp et al. 1997, 2005; Allsopp & Allsopp 2001, 2007). Recombination is likely to occur when either the mammalian or the vertebrate host is infected with more than one isolate, or two infected ticks with two different isolates feed on the same animal. Double infections have been demonstrated before in the ruminant host, for example, the Kümm isolate, where two distinct genotypes were isolated from one blood sample, as described previously by Zweygarth et al. (2002). However, it is also likely that recombination occurs in the tick during feeding where the tick is already infected with a strain from a previous feeding on a different infected animal (Allsopp et al. 2007). Recombination was also observed in several field samples. Nakao et al. (2011) found recombination between known and unknown strains in Uganda similar to the recombination between known strains that were described in SA (Allsopp et al. 2007; Aguiar et al. 2013; Frutos et al. 2007).

This study reports on a molecular epidemiological study of *E. ruminantium* in field samples from SA that were collected from different heartwater-endemic areas. This was done to characterise field isolates and confirm genetic diversity within *E. ruminantium* in SA. This study focussed on comparison of the conserved pCS20 gene region in different isolates. The pCS20 gene region is conserved and useful for the isolation of *E. ruminantium* in an epidemiological study but is not polymorphic enough to distinguish between new isolates that clustered together in the pCS20 phylogenetic tree. For example, the pCS20 region could not differentiate between the Welgevonden stock and the Ball3 strain that was used to develop the blood vaccine. Therefore, four polymorphic open reading frames (ORFs) (Erum0250, Erum1290, Erum6300 and Erum8340) were chosen for in-depth genotype characterisation. These ORFs were previously identified by subtraction hybridisation using *E. ruminantium* DNA from Welgevonden and Senegal stocks (Unpublished). They are highly polymorphic and have an identity that varies between 66% and 92%. The ORFs showed homology to unknown proteins or had no significant similarity to known sequences in the public databases. Eight of these stocks were further characterised using the small-subunit 16S rRNA gene to confirm that all the samples detected and used as reference strains belonged to *E. ruminantium* species.

## Materials and methods

### Study area

This study was conducted in heartwater-endemic areas of SA where the vector tick (*A. hebraeum*) occurs. A total of 3792 samples (blood, *n* = 2385; brain, *n* = 4; and *A. hebraeum* ticks, *n* = 1403) were collected from randomly selected cattle, sheep and goats from 122 selected farms and rural areas including communal dip tanks (Figure 1). Samples were collected using the random geographic coordinate method (Cameron 1999). A geographic area where heartwater occurs was first divided into districts. Farms were randomly selected in districts with a high mortality rate because of heartwater or where continuous problems with the disease were reported. This included the Eastern Cape (EC) (Grahamstown, Bathurst, Port Alfred, Alexandria and East London districts), KwaZulu-Natal (Vryheid, Pongola, Paulpietersburg, Umzinto, Mzinga, Hluhluwe, Ladysmith, Bulwer and Ulundi districts), Mpumalanga (MP) (Kruger National Park, Nelspruit, Witrivier, Barberton and Lydenburg districts), Limpopo (LM) (Potgietersrus district, and on a farm Springbokfontein [SBF] in the Tolwe District where a heartwater vaccine trial was also conducted), North West (NW) Province (Rustenburg District) and Gauteng (GP) (Roodeplaat and Pretoria-North districts) (Figure 1). Samples (*n* = 40) were collected as negative controls in the Free State (Warden district) where the tick vector does not occur. In all of these cases, the disease status of the animals was unknown.

**FIGURE 1:**
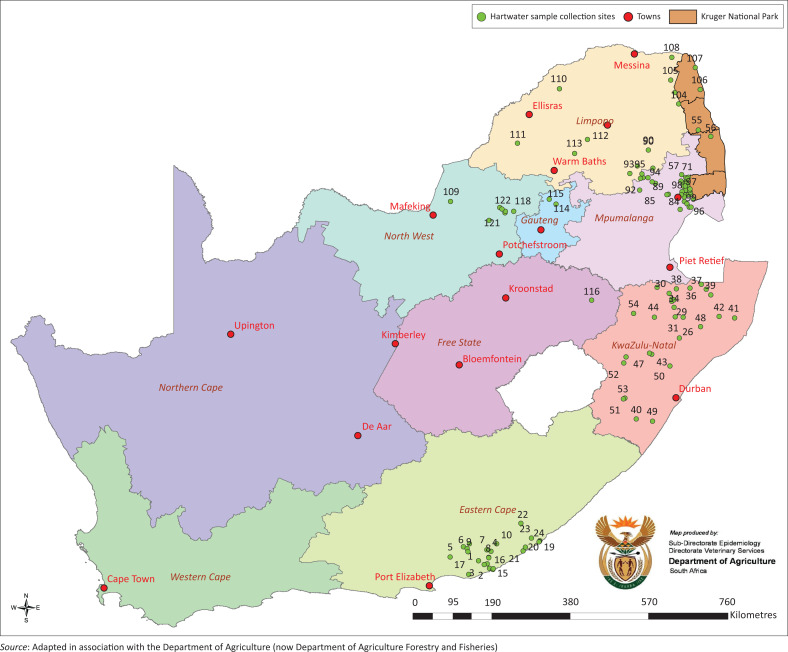
A map of South Africa indicating the commercial farms and dip tanks in rural areas used to collect blood and tick (*Amblyomma hebraeum*) samples for characterisation of *Ehrlichia ruminantium* in the endemic regions of South Africa. The heartwater sample collection sites are indicated with green dots.

### Samples and extraction of deoxyribonucleic acid

Blood from cattle was collected from the coccygeal vein, while in sheep and goats it was collected from the jugular vein, into sterile heparin Vacutainer^®^ tubes. The local veterinarian who did the post-mortem collected brain tissue samples from four animals (two bovines, one sheep and one goat), and they were kept at -20 °C until used. Ticks were collected from the same animals that were bled in the field or by drag sampling as described previously (Horak et al. 2006). Each live tick was washed with 70% ethanol, rinsed in distilled water, dried with tissue paper and cut in two and homogenised in 1.5 mL sucrose potassium glutamate (SPG), and the homogenate was stored at -20 °C until used. Genomic DNA was extracted from whole blood (200 *µ*L) or homogenised tick extract (200 *µ*L) using the QIAamp^®^ DNA Mini kit (QIAgen) according to the manufacturers’ instructions and was stored at -20 °C until further use.

### Phylogenetic screening and analyses using deoxyribonucleic acid sequence of the pCS20 region, 16S ribosomal ribonucleic acid gene and four polymorphic genes

The standard pCS20 polymerase chain reaction (PCR) diagnostic assays, PCR/probe and quantitative polymerase chain reaction (qPCR) (TaqMan) probe, were performed as described (Steyn et al. 2008; Van Heerden et al. 2004) and were used to screen the collected blood and tick samples for the presence of *E. ruminantium*. Samples that tested positive were further analysed by amplifying the pCS20 region using a PCR method previously published (Van Heerden et al. 2004) with the HH1F and HH2R primers (Table 1-A1) that gave a 720 bp amplicon. The small-subunit 16S rRNA gene method described by Allsopp et al. (1997) was used to confirm that all the samples detected and used as reference strains belonged to *E. ruminantium*. Four polymorphic ORFs, Erum0250, Erum1290, Erum6300 and Erum8340, previously identified using subtractive hybridisation assays (Unpublished) were selected for analysis. Primer sets for these ORFs were designed for PCR and sequencing (IDT Whitehead Sci.) (Table 1-A1) and were used to amplify all pCS20-positive samples and all known strains. Positive (Welgevonden genomic DNA) and negative (no genomic DNA) controls were included in each run.

Four 25 *µ*L PCR reactions of each sample amplified with each ORF primer set were pooled after amplification. The pooled amplicons were purified with MSB^®^ Spin PCRapace kit (STRATEC Molecular, Berlin, Germany) and sequenced with the primers from each amplicon. Sequencing was done using the BigDye Terminator version 3.1 cycle sequencing kit (Applied Biosystems) on an ABI 3100 sequencer (Applied Biosystems), and kits were used as recommended by the manufacturer. Each amplicon was sequenced three times to obtain a consensus sequence. Internal primers were required for the sequencing of the two largest ORFs: Erum1290 and Erum8340. Sequences of each sample in an ORF were cut to the same length. Sequence alignment as well as phylogenetic trees were inferred and visualised using the QIAgen CLC Genomics Workbench version 9 (http://www.clcbio.com/products/clc-genomics-workbench/). All the trees were unrooted.

### Culturing and characterisation of field isolates

Blood stabilates were prepared in 10 mL aliquots of blood collected from animals in the field as described previously (Brayton et al. 2003). Briefly, infected blood (5 mL) was mixed with SPG (5 mL) (Sucrose, 0.218 M; KH_2_PO4, 0.0038 M; K_2_HPO4, 0.0072 M; and glutamate, 0.0049 M; Bovarnick, Miller & Snyder 1950). These stabilates were stored in liquid nitrogen until used. The blood stabilate was thawed at 25 °C in water diluted 1:300 in SPG and 10 mL were used to infect a sheep within 20 minutes by the intravenous (IV) route (Brayton et al. 2003). A total of nine naïve 8-month-old Merino sheep obtained from heartwater-free areas were used in this trial. Two blood stabilate samples used were collected from the current study: (1) blood stabilate from a sick Angora goat (2 mL) from the farm Riverside (EC Province); and (2) blood stabilate from a sick sheep (10 mL) from the farm Grootvallei in the Potgietersrus District (LM province). In addition to these, six naïve sheep were each infected with six different blood stabilates from sheep that died of heartwater during a vaccine trial at the SBF farm (named as SBF1, SBF2, SBF4, SBF5, SBF6 and SBF7) (Collins et al. 2003; Pretorius et al. 2008). One sheep was infected with the Omatjenne isolate (10 mL) collected from Namibia (Du Plessis 1990). The Omatjenne isolate was not previously propagated in cell cultures but was included in this study to serve as a reference strain. The temperatures of the infected sheep were observed daily after inoculation, and the animals were monitored for clinical signs of heartwater, which include heavy breathing and loss of appetite, depression, hanging head, stiff gait, excessive chewing movement and nervous signs. Blood was collected from the jugular vein into sterile heparin Vacutainer^®^ tubes 3 days after an increase in temperature above 41.5 °C was observed for *in vitro* culturing, and the sheep were treated thereafter with Terramycin^®^100. Blood collected at the peak infection was used to initiate cell cultures for further characterisation as described previously (Zweygarth & Josemans 2001). Once the cultures were established, the isolates were characterised using the pCS20 region (method described above), and these isolates were named reference strains. The small-subunit 16S rRNA gene (Allsopp et al. 1997) was used to confirm that all the samples detected and used as reference strains belonged to *E. ruminantium*. The 16S rRNA gene was amplified and sequenced with vector-specified primers, T7 Forward and SP6 Reverse (Table 1-A1), and these amplicons were cloned in pGEM-T easy vector (Promega, USA) and transformed into TOP10 (Invitogen^®^) electrocompetent cells. Plasmids with the correct-sized inserts were sequenced three times as described above and the consensus sequences of each were analysed.

### GenBank accession numbers

The GenBank nucleotide accession numbers of new strains and uncultured isolates within the pCS20 gene region, Erum0250, Erum1290, Erum6300 and Erum8340 ORFs, and the 16S rRNA gene were submitted to GenBank (Table 2-A1).

### Ethical considerations

All animal work was performed in accordance with the stipulation of the animal ethics committee at the Agricultural Research Council – Onderstepoort Veterinary Institute Animal Ethics Committee (ethical clearance number: 9/15/p001). The animal ethics was approved 2006/2007 on project numbers: Department of Agriculture OV9/23/C167 grant, the FP6 EU INEV EPIGENEVAC FP6-003713 grant and the Red Meat Research and Development Trust of South Africa.

## Results and discussion

### Screening of field samples with the pCS20 quantitative polymerase chain reaction (TaqMan) probe assay

The field samples collected in this study included blood, brain samples and *A. hebraeum* ticks obtained from cattle, sheep and goats (Table 1). From 3788 field samples, 25% tick (344/1403) and 3.5% (85/2381) blood samples tested positive with the pCS20 assay. The observed low numbers of blood samples that tested positive for *E. ruminantium* could be attributed to the disease state of the animals. Previous experiments have shown that the pCS20 test can only detect high levels of *E. ruminantium* in blood that develop one day before the onset of and during the febrile reaction (Steyn et al. 2008). Most of the ticks collected from the animals (data not shown) were positive while the blood sample collected from the same animal tested negative. It is possible that the ticks were collected prior to the febrile reaction and the pathogen could not yet be detected in the blood. Other possibilities to consider are that the animals were immune to heartwater because of endemic stability (repeated exposure). In addition, the farmers regularly treat their animals with antibiotics, especially the Angora goat farmers, if they appeared sick or depressed. In spite of this, two sick animals were found in the field, one Angora goat from the Riverside farm in the EC and one sheep from the farm Grootvallei in the LM Province that died from heartwater. Two bovine brain samples were sent in from the NW Province to confirm heartwater. These all tested positive with the pCS20 TaqMan probe assay.

**TABLE 1:** The combined results for pCS20-positive samples collected from cattle, sheep, goats and ticks in the six heartwater-endemic regions of South Africa.

Samples	Source	Area collected (number positive/total number tested)						Total
		MP	LM	KZN	EC	NW	GP	
**Blood**	Cattle/	10/1101	3/4	16/29	16/248	3/74	-	48/1723
	sheep/	-	6/19	10/178	1/134	-	3/19	20/350
	goat	2/21	-	9/242	6/45	-	-	17/308
**Ticks**	Cattle/sheep/goat	130/505	72/207	51/167	85/332	6/192	-	344/1403
**Brain**	Sheep/goat	-	1/1(s)	-	1/1(g)	2/2(b)	-	4/4

MP, Mpumalanga; LM, Limpopo; KZN, KwaZulu-Natal; EC, Eastern Cape; NW, North West; GP, Gauteng; s, sheep; g, goat; b, bovine.

### Characterisation of field strains using pCS20

**FIGURE 2:**
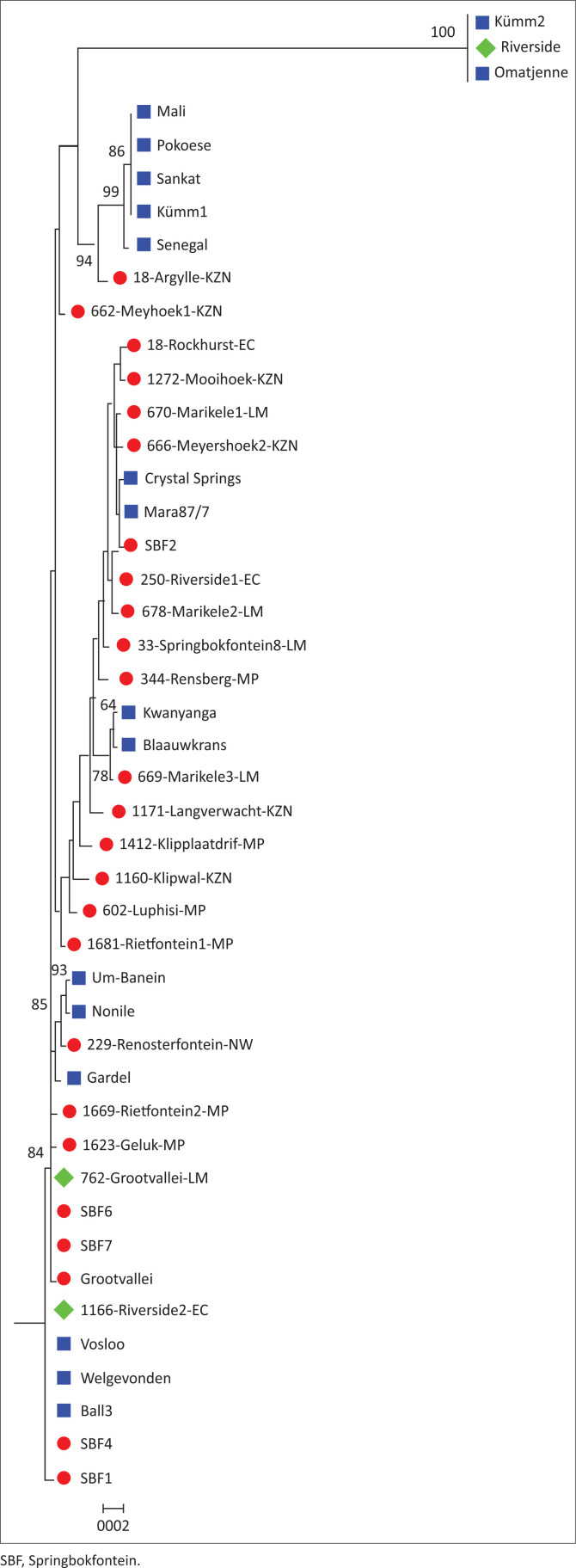
An unrooted phylogenetic tree of collected field isolates using the pCS20 gene region. The tree was constructed using the reference (blue) cultured strains and representatives of new isolates (red) in South Africa, with a neighbour-joining maximum composite likelihood approach. Propagated new stocks are indicated in green.

From the field samples (blood and ticks, *n* = 3792) that originated from different geographical sites (122 farms) in SA (Figure 1), only 433 blood and tick samples tested positive with the pCS20 assay. Of these, only 167 field samples could be successfully sequenced and used to infer a pCS20 gene region maximum likelihood unrooted tree (Figure 1-A1). In order to simplify this tree, identical sequences from the same farm were represented by a single sequence, and all new sequences were compared to the 17 known reference strains (Figure 2). A detailed description of all the sites these samples were collected from is listed in Table 3-A1. Even though the pCS20 region is conserved (Collins et al. 2003) and is used for heartwater diagnosis (Steyn et al. 2008), it was still possible to distinguish 25 novel clusters in our study.

The strains and field isolates are consistently separated into two major clades: the Welgevonden (SA) and the East and West Africa clades. The overall South- and West African clustering is similar to other studies *albeit* using different genes including *map*1 and other housekeeping genes (Adakal et al. 2009; Allsopp et al. 2001, 2007, 2009; Barbet et al. 2009; Faburay et al. 2008; Martinez et al. 2004; Nakao et al. 2011). Similar to pCS20, the *map*1 gene family is also separated into these two major clades (Allsopp et al. 2001). The *map*1 family is conserved between strains (98% – 99%) except for *map*1-2 paralog with a diversity of 79% (Barbet et al. 2009). In most studies on *map*1, Chrystal-Springs and Mara87/7 cluster together, but were not identical (Allsopp et al. 2001). In the current pCS20 analysis, we could not detect any sequence difference between these two strains. Although not identical in sequence, Welgevonden and the Nonile strains clustered together in the *map*1 gene analysis. In the current pCS20 analysis, Nonile and Um-Banein were identical and clustered together with Gardel. This was also the case using the16S rRNA gene. Senegal and Kümm1 clustered together in both genes tested in our study.

Furthermore, this study could define heterogeneity of strains in different locations in SA, as more than one isolate was found in an area and even on a farm. For example, nine field isolates that clustered together with the Welgevonden, Ball3 and Vosloo strains were geographically distributed in four provinces (three LM, two MP, three EC and one NW) on seven different farms (Figure 2). This is similar to previous findings using *E. ruminantium* and other *Ehrlichia* spp. Different genotyping methods of field samples collected in SA, Africa and Caribbean islands, using the *map*1 gene family, housekeeping genes or the pCS20 gene region, indicated that there is no geographical pattern on the distribution of *E. ruminantium* (Allsopp et al. 2001, 2007; Pilet et al. 2012). *Ehrlichia chaffeensis* isolates from different geographic areas in the United States showed no diversity between them (Yu, McBride & Walker 2007), while in some cases, isolates from different geographic areas belonged to the same group (i.e. from Tennessee, Florida and Arkansas). Comparison amongst various isolates using the outer membrane protein of *E. chaffeensis p28-19* gene revealed that heterogeneity exists between isolates. This *p28* gene belongs to the pfam01617 protein family and is present in *E. chaffeensis, Ehrlichia canis, Ehrlichia ewingii, E. ruminantium* and *Ehrlichia muris* (Yu et al. 2007). This gene sequence showed more diversity, and it is present throughout the coding sequences.

### Culturing of new field isolates

Naïve sheep were infected with the Omatjenne, 758-GVL-LM, 1166-RVE-EC and the SBF 1–7 isolates for propagation in cell cultures. The animals showed clinical signs of heartwater of heavy breathing and loss of appetite, depression, hanging head, stiff gait, excessive chewing movement and nervous symptoms. All the sheep had a temperature of 42 °C for 3 days and were bled on the third day of febrile reaction and treatment was administered for 3 days. Interestingly, the sheep infected with the Omatjenne isolate showed identical heartwater signs. The new isolate names, origin and type of cell cultures used for propagation are listed in Table 2.

**TABLE 2:** The nine new isolates established in cell culture.

Name	Original field samples	Animal no.	Established in the following cell line(s)
SBF1	Goat 24 from SBF	Sheep 432	BA
SBF2	Tick from SBF	Bovine 9331/0	BA
SBF4	OVI HW experiment sheep 817	Sheep 851	IDE8 tick
SBF5	OVI HW experiment sheep 828	Sheep 859	BA and IDE8
SBF6	OVI HW experiment sheep 830	Sheep 872	BA
SBF7	OVI HW experiment sheep 837	Sheep 933	BA
Grootvallei	Sheep 6/316	Sheep 863	BA
Omatjenne	Blood stabilate	Sheep 6000	E_2_ and IDE8
Riverside	Angora goat from Eastern Cape	Sheep 849	IDE8

SBF, Springbokfontein; OVI, Onderstepoort Veterinary Institute; HW, Heartwater; BAE, Bovine aorta endothelial; IDE8, Ixodes scapularis-derived cell line 8.

Six blood isolates propagated in cell cultures from the LM province originated from the SBF farm. These blood isolates from the SBF farm were positive for *E. ruminantium* with the pCS20 assay before and after they were propagated in cell cultures confirming that these strains were the same as the original sequences. The SBF farm was used for a DNA vaccine experimental field trial (Pretorius et al. 2008), and six novel strains were found on this farm as well as 38 other isolates (Figure 1-A1). In total, nine new strains were propagated in cell culture and were sequenced to confirm that the sequence was the same as the original blood sample. Three new strains SBF6, SBF7 and Grootvallei and 59 field genotypes (Figure 1-A1) from other areas clustered together in this new cluster that was closely related to Welgevonden strain but differ with two base pairs. Two SBF isolates, four new SBF strains and 10 field isolates were identical to Welgevonden. Two new strains SBF2 and SBF5 and 34 other field isolates clustered with Mara87/7. A new cluster formed close to Welgevonden and originated from the 758-GLV-LM (Grootvallei farm, Potgietersrus) and SBF farm (SBF6 and SBF7) and both were in the LM province. The Welgevonden, Ball3 and Vosloo strains cluster together with two new strains (SBF1 and SBF4) from the SBF farm. The Um-Banein (Sudan) and Nonile (KwaZulu-Natal [KZN]) cluster together. However, Kümm2, Omatjenne and 1166-RVE-EC (Riverside farm) clustered on their own and had an identity similarity between 81% with the rest of the *E. ruminantium* sequences.

The Riverside strain named after the farm was isolated from an Angora goat kid from the EC. The original blood sample (1166-RVE-EC) used for sequencing and phylogenetic analysis was identical to the Welgevonden isolate. A Merino sheep was subsequently infected with the goat blood stabilate at the ARC-OVI to establish this isolate in cell culture (IDE8 cells). However, after propagation in cell culture, the sequence was identical to the Kümm2 strain and the original isolate was lost. It could be that there was more than one *E. ruminantium* isolate in the original goat blood sample. Similar results were obtained by Zweygarth et al. (2002). They could distinguish between the two isolates, Kümm1 and Kümm2, by culturing the Kümm strain in different cell lines using BA cells and the IDE8 tick cell line. One of the isolates preferred growth in the IDE8 cells, while the other was suppressed. The opposite was also confirmed where it was shown that the Welgevonden strain grows in the BA cells and Kümm2 does not (Zweygarth et al. 2002). Therefore, it was possible that the original Riverside sample was infected with two isolates and at least one of these preferred growth in the IDE8 tick cell line. The known Kümm1 strain is the only South African strain that clustered together with the West African group but is not identical to them. Interestingly, two field isolates from the EC and two from two farms in KZN grouped closely together with the Western African cluster. It is the first time that we have found isolates that are close to the West Africa clade.

### 16S ribosomal ribonucleic acid gene

**FIGURE 3:**
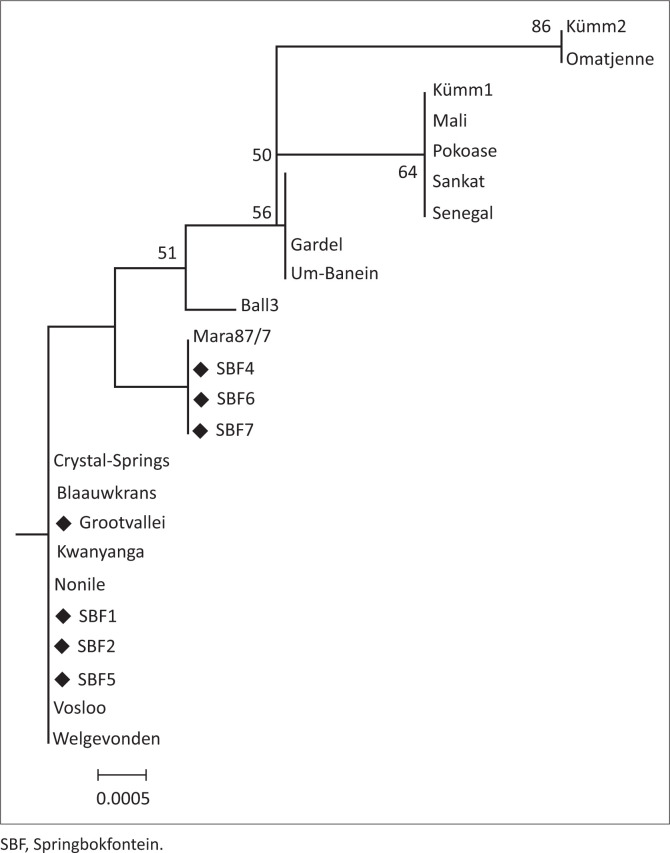
An unrooted phylogenetic tree was constructed using the 16S ribosomal ribonucleic acid sequence comparing the newly cultured strains (diamonds) with the reference strains in South Africa using a neighbour-joining maximum composite likelihood approach.

The 16S rRNA gene sequence was used to confirm that the new isolates were that of *E. ruminantium*. Results from the phylogenetic tree confirmed that the new strains belonged to the *E. ruminantium* species (Figure 3). Three of the new SBF (4, 6 and 7) strains clustered with Mara87/7 and Grootvallei, and the three SBF (1, 2 and 5) strains clustered with Welgevonden. None of the new strains clustered with the Western African clade except for the old Kümm1 strain. The Um-Banein and the Gardel strains were closely related within the pCS20 gene regions with only five nucleotide difference; we found that these two strains also grouped together using the 16S rRNA V1 loop gene region. The Kümm2 and Omatjenne strains clustered on their own in the 16S rRNA gene but closer to the West Africa strains, and in the pCS20 gene region they cluster on their own but closer to the West Africa and Mara/Blaauwkrans group. The Kümm2 and Omatjenne strains were previously described by Allsopp and Allsopp (2007) as non-pathogenic strains that cluster closely to *E. ruminantium* that was isolated from Namibia (Du Plessis & Kümm 1971). Interestingly, the Omatjenne was isolated from a *Hyalomma truncatum* adult tick (Du Plessis 1990) and that could explain why it differs from *E. ruminantium*. Although, it fits in the *E. ruminantium* group and potentially has the same clinical symptoms as heartwater, further in-depth characterisation is required. This may be done by genome sequencing of the Kümm and Omatjenne strains determining their host tick species and evaluating their pathogenicity in ruminants in order to reclassify them.

### Phylogenetic analyses using deoxyribonucleic acid sequence of four polymorphic genes

**FIGURE 4:**
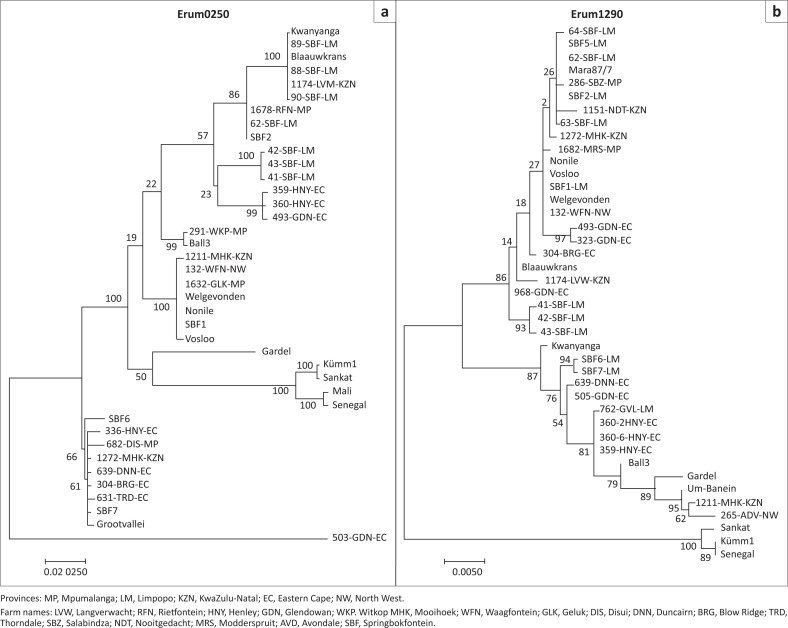
Phylogenetic tree constructed (a) from Erum0250 deoxyribonucleic acid sequence data of 11 reference strains and 25 field isolates and (b) from Erum1290 deoxyribonucleic acid sequence data of 16 *Ehrlichia ruminantium* reference strains, seven new strains and 33 field isolates from blood and ticks in the heartwater-endemic regions of South Africa.

**FIGURE 5:**
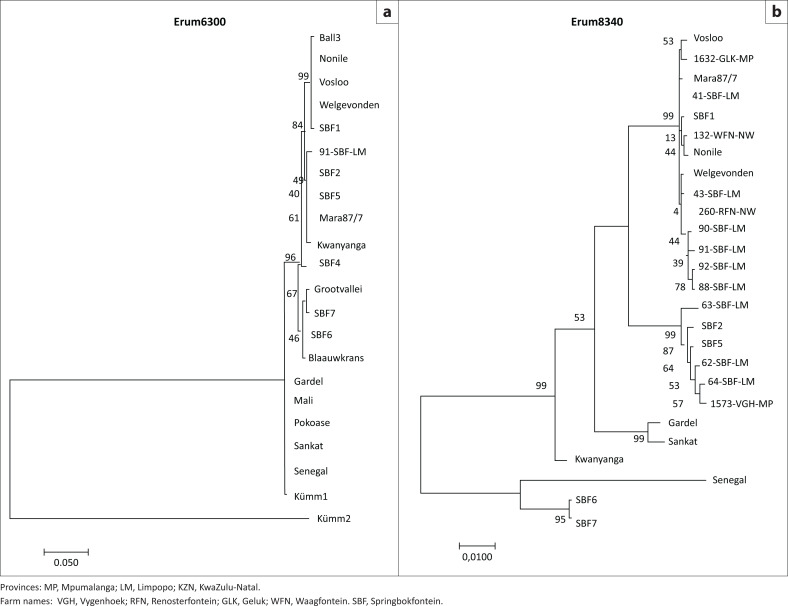
Phylogenetic tree constructed (a) from Erum6300 data of 16 *Ehrlichia ruminantium* reference strains, seven new strains and one field isolate and (b) from Erum8340 nucleic acid sequence of 16 *E. ruminantium* reference strains, seven new strains and 13 field isolates collected from blood and ticks in the heartwater-endemic regions of South Africa.

The four polymorphic ORFs (Erum6300, 8340, 0250 and 1290) had different polymorphism profiles (Figures 4 and 5). Erum6300 divided the known strains into clusters that were similar to the pCS20 clusters, and only one field strain could be sequenced. However, with the Erum8340, more field isolates could be distinguished from each other while known strains (Grootvallei and SBF4) could not be sequenced. With the Erum0250 and Erum1290, more field isolates and known strains could be distinguished from each other. This indicates that even if the strains had identical pCS20 sequences, they however do differ from each other, confirming the huge genetic diversity in the field.

## Conclusion

The current study identified 25 new *E. ruminantium* clusters based on pCS20 data. In addition, nine new isolates were propagated in cell culture. This study showed that there is major genetic diversity in SA, and this knowledge is essential for control of heartwater and for vaccine development. The impact of moving animals from one endemic area to another could increase recombination of strains to more virulent ones. Thus, continuous epidemiological studies are needed to identify and grow isolates *in vitro* for further characterisation. As recombination has been shown to occur in many genes, future epidemiological studies should focus on complete genome sequencing of all cultured isolates. In addition, the cross-protection status of newly cultured isolates should be determined and compared with the highly virulent Welgevonden strain, as we currently base all heartwater vaccine studies on this strain.

## Figures and Tables

**FIGURE 1-A1:**
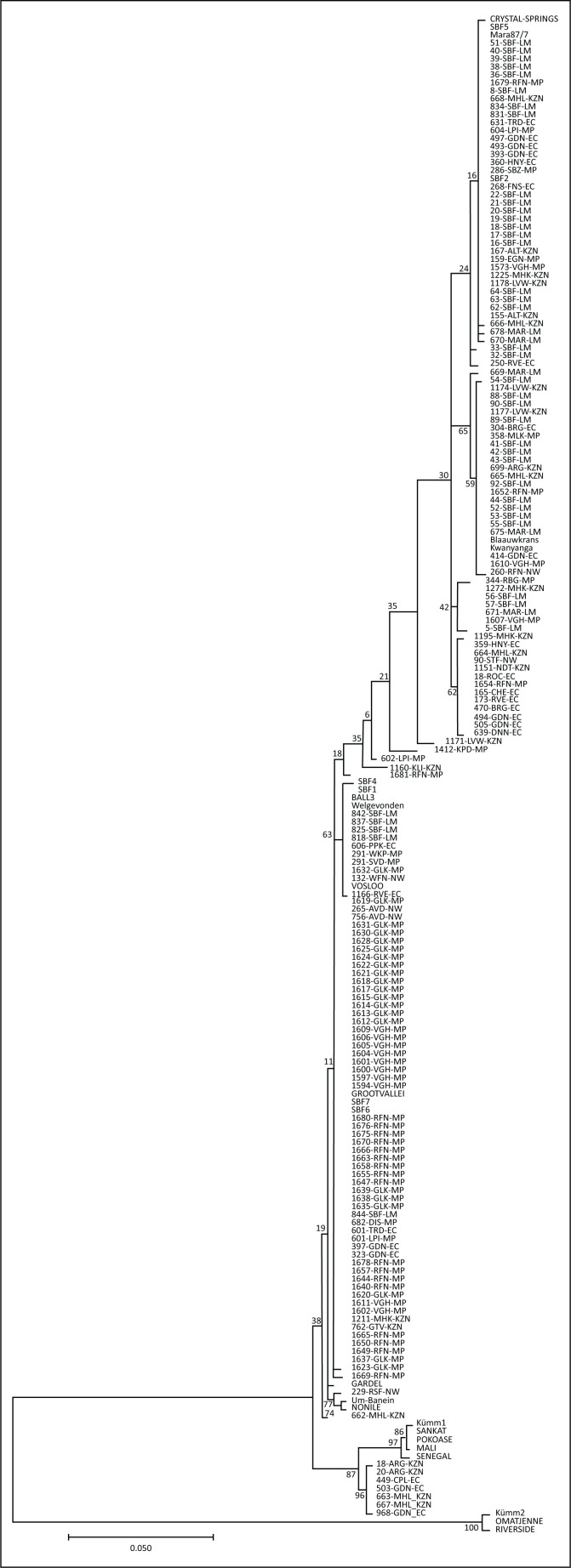
An unrooted phylogenetic tree of all collected field isolates and reference strains using the pCS20 gene region. The tree was constructed with a neighbor-joining Maximum Composite Likelihood (MCL) approach. The data is summarised in Figure 2.

## Appendix 1

**TABLE 1-A1:** Primer sequences used to amplify and sequence the, Erum0250, Erum1290, Erum6300, Erum8340 ORFs, HH1F, HH2R, 16sF and 16sR, CowF, CowR and CowTM probe.

Primer Name	Sequence 5′ - 3′	T(a)^1^°C	Base pares
Erum0250 Forward	GGA TCC ATG GGT ATT GAT AA TTA TG	52	1455
Erum0250 Reverse	TCT AGA AGC AAA TGT AAT TTC ATG		
Erum1290 Forward	GGA TCC ATG AAA GAA AAT ATA GAA C	58	1560
Erum1290 Reverse	TCT AGA CTA TTG TAA TAG CCC AAA G		
Erum1290 Forward Internal	CAT TAT CAT TCT ACA ACA AAT GAC AAG		
Erum6300 Forward	GGA TCC ATG CAG TGA AAG TAG G	58	930
Erum8340 Reverse	TCT AGA TTA TCT ACC ACA TCC TTT ATT TC		
Erum8340 Forward Internal	CAT GAA ATA CCT CAG CCA AGC GG	60	1670
Erum8340 Reverse Internal	GTA CAA ACA ATT CTT TCA ATG C		
HH1F	CCC TAT GAT ACA GAA GGT AAC CTC GC	62	900
HH2R	GAT AAG GAG ATA ACG TTT GTT TGG		
Cow Forward primer	CAA AAC TAG TAG AAA TTG CAC A	58	187
Cow Reverse primer	TGC ATC TTG TGG TGG TAC		
CowTM probe	6FAM TCC TCC ATC AAG ATA TAT AGC ACC TAT TA XT-PH		
T7	CGA ACT CAT AAG ATA TCA CAG TGG ATT TA	50	-
SP6	GTA ATA CGA CTC ACT ATA GGG C		

T(a), Annealing temperature.

**TABLE 2-A1:** GenBank Accession numbers of the 16Sr RNA and PCS20 gene region.

Gene ID	GenBank Accession nr.
16S: new stocks	KF786042 - KF786047
pCS20 new stocks	KF786113 - KF786120
pCS20 uncultured	KF786204 – KF786288
Erum0250 cultured	KF786049 - KF786064 (20)
Erum0250 uncultured	KF786205 - KF786226 (22)
Erum6300 cultured	KF786065 - KF786085 (21)
Erum6300 uncultured	KF786086 (1)
Erum8340 cultured	KF786087 - KF786099 (13)
Erum8340 uncultured	KF786100 - KF786112 (13)
BankIt1665159:pCS20 cultured	KF786113 - KF786120 (8)
BankIt1665207:pCS20 uncultured	KF786121 - KF786204 (84)
BankIt1669234:Erum1290 uncultured	KF786247 - KF786279 (33)
BankIt1669230:Erum1290 cultured	KF786227 - KF786246 (20)

**TABLE 3-A1:** A summary of the samples collected in South Africa.

Isolate specifications	Reference Strains in culture	New Genotype	Origin	Specie	Sample type	Farm name/s	Province	Nr. Genotype identical
Groups	Kümm2/Omatjenne	-	SA	-	-	-	-	1
	-	Riverside	SA	Angora goat	Blood	Riverside	EC	-
	Mali	-	WA	-	-	-	-	0
West Africa Group	Pokoase	-	WA	-	-	-	-	0
	Sankat	-	WA	-	-	-	-	0
	Senegal	-	WA	-	-	-	-	0
	Kümm1	-	SA	-	-	-	-	0
New Groups	-	18-Argylle	SA	*A. hebr*	Tick	4 Farms	KZN, EC	6
	-	662 Meyershoek1	SA	*A. hebr*	Tick	Meyershoek	KZN	0
	-	18 Rockhurst	SA	Sheep	Blood	Rockhurst	EC	13
	-	1272 Mooihoek	SA	*A. hebr*	Tick	Mooihoek	KZN	6
	-	344 Rensberg	-	*A. hebr*	Tick	Rensberg	MP	0
Mara Group	-	670 Marikele1	SA	Buffalo	Blood	Marikele	LM	0
	-	666 Meyershoek2	SA	*A. hebr*	Tick	Meyershoek	KZN	-
	Chrystal-Springs	-	ZIM	-	-	-	-	31
	Mara87/7	SBF2	SA	Sheep	Blood	12 Farms	6 Prov	-
	-	250-Riverside	SA	Angora goat	Blood	Riverside, SBF	EC, SBF	2
	-	678 Marikele2	SA	Buffalo	Blood	Marikele	LM	0
	-	33 SBF8	SA	*A. hebr*	Tick	SBF	LM	0
	-	344 Rensberg	SA	*A. hebr*	Tick	Rensberg	MP	0
Kwan/Blaauw Group	Kwanyanga/Blaauwkrans	-	SA	-	Bl/Tick	10 farms	4 Prov	25
	-	669 Marikele3	SA	Buffalo	Blood	Marikele	LM	0
	-	1171 Langverwacht	SA	*A. hebr*	Tick	Langverwacht	KZN	0
	-	1412 Klipplaatdrif	SA	*A. hebr*	Tick	Klipplaatdrif	MP	0
	-	1160 Klipwal	SA	*A. hebr*	Tick	Klipwal	KZN	0
	-	602 Luphisi	SA	*A. hebr*	Tick	Luphisi	MP	0
	-	1681 Rietfontein1	SA	*A. hebr*	Tick	Rietfontein	MP	0
Welg Group	Um-Banein/Nonile	-	EA/SA	*-*	-	-	-	0
	-	229 Renosterfontein	SA	*A. hebr*	Tick	-	-	0
	Gardel	-	WA	*-*	-	--	-	0
	-	1669 Rietfontein2	SA	*A. hebr*	Tick	-	MP	0
	-	1623 Geluk	SA	*A. hebr*	Tick	-	MP	0
	-	Grootvallei	SA	Sheep	Blood	12 Farms	5 Prov	58
	-	SBF6	SA	Sheep	Blood	SBF	LM	-
	-	SBF7	SA	Sheep	Blood	SBF	LM	-
	Welgevonden/Ball3/Vosloo	SBF1/SBF4	SA	Sheep	Blood	SBF	LM	15

SA, South Africa; WA, West Africa; EA, East Africa; ZIM, Zimbabwe; Prov, Province; *A. hebr, Amblyomma hebraeum*; EC, Eastern Cape; KZN, KwaZulu-Natal; MP, Mpumalanga; LM, Limpopo; NW, North West; SBF, Springbokfontein.
